# Nitrocellulose Based Film-Forming Gels with Cinnamon Essential Oil for Covering Surface Wounds

**DOI:** 10.3390/polym15041057

**Published:** 2023-02-20

**Authors:** Lauryna Pudžiuvelytė, Evelina Drulytė, Jurga Bernatonienė

**Affiliations:** 1 Institute of Pharmaceutical Technologies, Medical Academy, Lithuanian University of Health Sciences, Sukileliu pr. 13, LT-50161 Kaunas, Lithuania; 2 Department of Drug Technology and Social Pharmacy, Medical Academy, Lithuanian University of Health Sciences, Sukileliu pr. 13, LT-50161 Kaunas, Lithuania

**Keywords:** nitrocellulose, film-forming gel, film, cinnamon leaf essential oil, wounds

## Abstract

Acute and chronic wounds caused by assorted reasons impact patient’s quality of life. Films are one of the main types of moisture retentive dressings for wounds. To improve the healing of the wound, films must ensure there is no microorganism contamination, protect from negative environmental effects, and support optimal moisture content. The aim of this study was to formulate optimal film-forming gel compositions that would have good physico-chemical properties and be suitable for wound treatment. Nitrocellulose, castor oil, ethanol (96%), ethyl acetate, and cinnamon leaf essential oil were used to create formulations. During the study, the drying rate, adhesion, flexibility, tensile strength, cohesiveness, swelling, water vapor penetration, pH value, and morphology properties of films were examined. Results showed that optimal concentrations of nitrocellulose for film-forming gel production were 13.4% and 15%. The concentrations of nitrocellulose and cinnamon leaf essential oil impacted the films’ physicochemical properties (drying rate, swelling, adhesion, flexibility, etc.). The swelling test showed that films of formulations could absorb significant amounts of simulant wound exudate. Film-forming gels and films showed no microbial contamination and were stable three months after production.

## 1. Introduction

A large part of society suffers from wounds caused by assorted reasons. They can be acute, formed by mechanical damage to the skin, injuries, and chronic, which are caused by certain diseases (diabetes, varicose, cancer, and others) and classified by etiology into four categories, each with its own typical location, depth, and appearance: arterial, diabetic, pressure, and venous [1,2,3]. Both types of wounds cause discomfort to patients: swelling, bleeding, exudation, purulence, non-healing, and infections; some chronic wounds can take decades to heal, thus contributing to secondary conditions such as depression, and can lead to isolation and family distress [2,3,4]. Semi-solid dosage forms such as creams, ointments, gels, and patches have traditionally been used to heal the wounds and deliver drug or natural active ingredients transdermally. However, the main disadvantage of these preparations is the limited contact with skin due to the rub-off by clothes during daily activities [5]. In this field, there is a need to obtain transdermal dosage forms which would avoid skin irritation and contact the skin closely for a prolonged time to deliver active compounds [5,6]. In this regard, the film forming system (FFS) is a novel approach which can be used as an alternative to conventional topical and transdermal formulations due to its strong adhesion to the skin, sustained drug delivery to the affected site, and the reduced need for frequent applications [6,7]. Film-forming gels are semi-solid formulations of both solid and liquid materials; usually transparent and colourless, they are cosmetically appealing [8]. A semi-solid form is obtained by treating the liquid phase with gelling agents, but the polymers must also have film-forming properties [9,10]. Film-forming gels form a thin, transparent film in situ by solvent evaporation [11]. Addition of certain materials into the composition of such gels allows to select various desired properties for the films, for example: easy application, high adherence to skin, resistance to water, air permeability, flexibility, the ability to absorb wound exudate and control its moisture, softness, and easy, painless removal of the films at the patient’s request [8,11]. It is also important that the resulting protective layer traps external pathogenic microorganisms, thus preventing them from penetrating the injured area and causing an infection [1]. The production of a film-forming gel with some or all of the above-mentioned properties would result in a dosage form optimally suited for protecting any wound from environmental factors and infection [8,11]. Natural substances used against infection could be essential oils [12]. Cinnamon (*Cinnamomum*, family *Lauraceae*) leaf or bark essential oils have various biological activities including antibacterial, antioxidant, antifungal, and cytotoxic [13,14]. Eugenol, benzyl benzoate, linalool, borneol cinnamaldehyde, trans-cinnamyl acetate, benzene dicarboxylic acid, α-pinene, and coumaric acid are the main chemical compounds determined in the composition of cinnamon essential oil [13,15].

The production of gels that form films, as found in the literature, is usually using the mass method. This production method is quite simple and does not require high technical costs. The liquid phase of film-forming gels is treated with gelling polymers capable of forming films, and other substances of significant importance to the effect of the drug are added after allowing the gel to form.

The aim of this work is to design nitrocellulose films containing cinnamon essential oil, to perform their optimization and to evaluate the physico-chemical, mechanical, and biopharmaceutical properties of the films.

## 2. Materials and Methods

### 2.1. Materials

The main ingredients used during experiments were 96% alcohol (AB “Vilniaus degtinė”, Vilnius, Lithuania), castor oil, ethyl acetate, sodium chloride, calcium chloride, buffer solution (pH = 7.5) (Sigma-Aldrich, Darmstadt, Germany), cinnamon leaf essential oil (UAB “Naujoji Barmunė”, Vilnius, Lithuania), jojoba oil (UAB “Kvapai”, Vilnius, Lithuania), purified water (Ph. Eur. 01/2008:0008, LSMU laboratory), gelatin (UAB “Klingai”, Vilnius, Lithuania).

### 2.2. Preparation of Gels Forming Films

The base of a film-forming gel was prepared from ethyl acetate, ethanol, and castor oil. Essential oil was mixed with ethanol, then ethyl acetate was added and mixed well again. The solution was mixed with castor oil. After stirring the mixture was clear and homogeneous. Then it was poured into a glass with a gelling agent—nitrocellulose and mixed again until a homogeneous consistency. The obtained product was poured into a container and sealed [1,6]. When the gel is spread on the coating surface and the films are formed, no cracks or wrinkles should be visible. The film must be colorless and transparent to be less noticeable and for the condition of the wound to be visible [16].

### 2.3. Evaluation of Film Drying Rate

A small amount of the gel was placed on a Parafilm at 33–36 °C and the time needed for the gel to form a film was recorded. If the film formed too quickly or too slowly, the formulation was adjusted [9]. After a drying time of 2 min, a glass plate was placed on the film and observed. If the film was dry there should be no liquid/gel residue on the plate. If the film was not dry, the test was repeated and the time for drying extended. A modified method was used to perform the test [6,16].

### 2.4. Assessment of Film Surface and Homogeneity Properties

Microscopy was used to evaluate the surface morphology of the formed films [17]. The films were cut into 3 × 5 mm^2^ pieces and placed on a microscope slide. Images were obtained using a microscope with a working distance of 15 mm and a magnification of 40×. No lumps or uneven surface should be visible during the test.

### 2.5. Assessment of Surface Adhesiveness and Wound Adhesion

The surface adhesion of films was determined when the obtained film was completely dry. A piece of cotton wool gently touched to its surface, should not stick to the film. Adhesion was considered high if a lot of cotton fibers remained, medium if only a few fibers remained, and the film was non-sticky if no fibers remained at all [6,9]. In addition to the above-mentioned method, there was another, more specific method for evaluating the adhesiveness of films: a texture analyzer (Stable Micro Systems Texture Analyzer TA. XT plus) was used. A completely dry piece of film measuring 2 × 2 cm^2^ was placed on a special fixed plastic table and pressed with a transparent plastic plate with a 1 cm diameter cavity in the middle, leaving an open part of the film. A stick with a flat end slightly less than 1 cm in diameter was attached to the metal support on top of the texture analyzer. The parameters were determined—the applied force during contact was 5 g, the contact time between the stick and the film—5 s, the speed of the stick movement during the test—1 mm/s. The texture analyzer lowered the stick until it pressed against the surface of the film, raised it up after 5 s and measured the force required to tear the end of the stick from the surface of the film.

The films prepared for adhesion test were cut into square strips (2 × 2 cm^2^) and attached to a probe with a diameter of 35 mm. Before the test, a simulation of wound was prepared from 20 g of 6.67% (V/V) gelatin in a 90 mm Petri dish and kept at 4 °C overnight. Gelatin surface was covered with 500 µL of an aqueous phosphate solution (pH = 7.5). The films were kept in contact with the gelatin for 1 min. The probe was set before and during the test for 0.5 mm/s speed, after the test speed was 1 mm/s with a force of 1 N. The peak adhesion force represents the maximum force required to remove the film from the simulated wound surface, the area under the curve (AUC) represents the overall adhesion, and the cohesion represents the distance (mm) to tear the film away from the wound [17].

### 2.6. Evaluation of Film Flexibility

The flexibility of the films was evaluated by resistance and tensile tests. A texture analyzer (Stable Micro Systems Texture Analyzer TA. XT plus) was used for both tests. Resistance testing was performed using a ball with a metal needle attached to a metal support. Film in 3 × 3 cm^2^ shape (n = 6 for each composition) was transferred to the middle of a special device stand with a round cavity of 1 cm diameter in the way that no wrinkles and unstressed areas were visible. The film was pressed so that the ball would not move and distort the results when hit it. The test parameters were determined—the ball’s downward pressure distance—40 mm. The test showed the force required to pierce the film, thus showing its flexibility. The tensile strength was calculated based on the formula [17]:

(1) $$\mathrm{Tensile}\;\mathrm{strength}\;(N/{\mathrm{mm}}^{2})=\frac{\mathrm{applied}\;\mathrm{force}\;\left(N\right)}{\mathrm{initial}\;\mathrm{cross}-\sec\mathrm{tional}\;\mathrm{area}\;\left({\mathrm{mm}}^{2}\right)}$$

A tensile test was performed, where the distance and force required to break the film was measured [1]. The tensile test of the films was performed with a texture analyzer by attaching films measuring 65 × 30 mm^2^ from both ends to clamps on opposite sides, approximately 3 cm apart (n = 6 for each composition). The clamps were held on a metal base. One of the clamps was locked, the other could move. During the measurement, the moving clamp pulls the film upwards. The stretching speed was 6 mm/s, the lifting distance of the moving clamp—150 mm. Tensile strength was measured when the film was torn [16,17].

### 2.7. Evaluation of Water Vapor Permeation and Swelling

The evaluation of water vapor permeation test was based on a modified Mu et al. method [1,18]. A round shape piece of film (n = 3 for each composition) was placed on a plastic tube of 30 mm diameter with 8 mL of distilled water. The tube was placed in an incubator, kept at a temperature of 38 ± 2 °C with a humidity of 60 ± 5% for 24 h. Water vapor transmission rate was calculated by the formula [19]:

(2) $$\mathrm{WVTR}=\frac{W/t}{A}$$

WVTR—water vapor transmission rate; it is the amount of water vapor penetrated in grams per square centimeter in 1 h interval (g cm^−2^ d^−1^)
W—change in weight (g),
t—time (d),
A—sample area (cm^2^).

Swelling was determined by weighing three 2 × 2 cm^2^ strips of film and immersing them in 10 mL of simulated wound fluid. Simulated wound fluid was prepared from 0.184 g of calcium chloride, 4.149 g of sodium chloride, and 1000 mL of purified water [20]. After films immersion in simulated wound fluid, changes in weight were observed every 15 min for 2 h. The hydrated films were gently blotted with filter paper to remove excess simulated wound fluid from the surface and then weighed again. The swelling index was calculated by the formula [17]:

(3) $$\mathrm{Swelling}\;\mathrm{index}\;\left(\%\right)=\frac{W_{s}-{\;W}_{d}}{W_{d}}\times 100$$

W_s_—the weight of the film after hydration,

W_d_—the weight of the film before hydration.

### 2.8. Microbiological Evaluation of Gel Quality

Microbiological evaluation of the gels was performed on 5, 5K, 6, and 6K samples. The physiological solution was dispensed into 5 mL individual tubes and used for preparation of suspension of the following bacteria: *Staphylococcus aureus* and *Pseudomonas aeruginosa*. All bacteria were isolated from clinical material. The broth liquid medium was dispensed into test tubes to give a final volume of 10 mL (with a sample of film-forming gel). The medium was sterilized. For each bacterial culture Mueller Hinton broth was used. The tubes were inoculated with 10 μL of bacterial suspension with the film-forming gel. After 48 h of incubation, each tube was inoculated with 10 μL of suspension on soy-tryptone agar (Thermo Fisher, Hampshire, UK) [21]. After this bacterial growth (bacterial colonies growing (+)/no growing (−)) was counted.

### 2.9. Determination of Stability and pH Value of Film-Forming Gels

The stability of gels forming films was determined after 1 month under accelerated conditions (equivalent to 3 months under normal conditions). The manufactured and packaged gels of the formulations 5 and 6 were stored at 38 ± 2 °C for 28 days. The appearance of films was recorded on the day of production, then on days 7, 14, 21, and 28 [22].

Meanwhile, the pH of the samples was determined immediately after gels preparation. Five grams of gel and 95 mL of purified water were put together, mixed for 10 min, filtered and the pH value measured [22].

### 2.10. Qualitative and Quantitative Evaluation of Essential Oil Composition of Cinnamon Leaves

For the qualitative and quantitative analysis of the essential oil of cinnamon leaves a Shimadzu GC-MS-QP2010 gas chromatograph-mass spectrometer system (Shimadzu, Tokyo, Japan) equipped with a Shimadzu autoinjector AOG-5000 (Shimadzu, Tokyo, Japan) was used. A capillary column RXI-5MS (30 m × 0.25 mm × 0.25 µm film thickness) was used. Sample of 0.2 µL was injected into the split/splitless injector with a split ratio 1:50. The operating conditions were as follows: the chamber temperature was maintained at 40 °C for 3 min and raised at 5 °C/min rate to 120 °C. After 3 min the temperature was reduced at 2 °C/min rate to 180 °C, maintained for 3 min. After 3 min the temperature was raised for the last time to 230 °C at 5 °C/min rate and again maintained for 3 min. Temperature of injector, detector and ion stream was 250 °C. Helium was used as the carrier gas. The identification of the components was based on the comparison of their mass spectrum with the spectra specified in the databases. Quantitative analysis of components was carried out by peak area normalization measurements [23].

### 2.11. Statistical Analysis

The results obtained in the tests were evaluated by statistical analysis. Data processing program IBM SPSS Statistics (version 27.0.1) (IBM Corporation, New York, NY, USA) was used to determine arithmetic means, standard deviations, and statistical significance. Significance was assessed by Kruskal-Wallis, one-way ANOVA (k samples) tests (significant when *p* < 0.05).

## 3. Results and Discussion

### 3.1. Effect of ingredients on Film-Forming Gel Formation and Appearance of Films

At the beginning of the study, empirical tests were conducted to find the optimal control composition. Table 1 shows that during the test, seven formulations and two controls with different amounts of used substances were produced, but only two met the visual and physical requirements. For further studies only two formulations were chosen.

**Figure 1:**
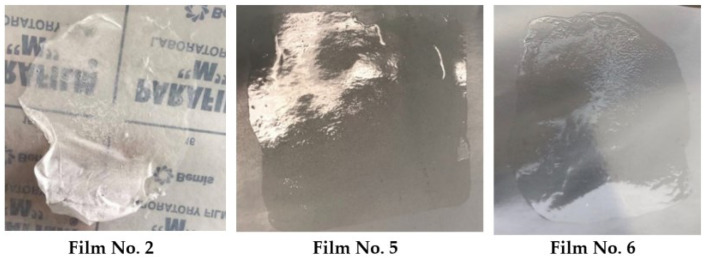
Appearance of films.

In the literature, concentration of cellulose derivatives in film-forming gels varied from 2% to 30% [24,25]. However, after making the product according to the first composition, which contained 11% nitrocellulose and 10% castor oil, the gel turned out liquid. The film formed, but the drying process was long, and the surface felt very sticky when dry. It also did not peel nicely from the Parafilm. Based on these observations, other formulations with higher amounts of nitrocellulose and lower amounts of castor oil were tested. By increasing the nitrocellulose content in the second composition to 15.4% and after reducing castor oil to 4% the gel was thicker, but the film was of white color. After it dried, large cracks appeared (Figure 1).

The visual investigation concluded that the film does not bend at all. This could be due to the smaller amount of castor oil. Increasing the castor oil content to 6.3% in formulation 3 produced a strong film that dried quickly and peeled off the surface, but the color remained off-white. The reason for that could be that the amount of nitrocellulose was too high. The same film appearance was obtained with formulation 4: 16% of nitrocellulose produced a thick gel, but the film was white, uneven, sticky, and cracked. Formulation 5 consisted of 13.4% nitrocellulose, the gel was significantly more liquid than in formulation 4, and it also formed transparent, elastic, fast-drying, and solid film (Figure 1).

However, stickiness was still noticeable, which can be annoying when in contact with clothes, so the amount of nitrocellulose in the composition was increased to 15%. Film 6 showed the best results—a colorless, fast-drying, a little sticky, solid, strong, but at the same time elastic film was formed (Figure 1).

**Figure 2:**
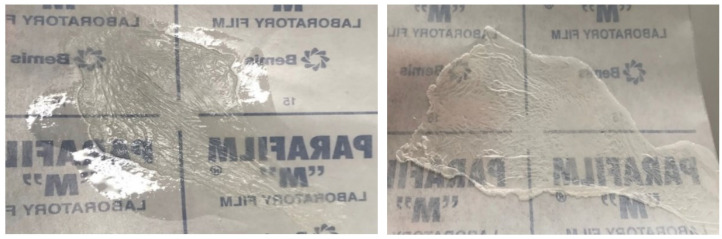
Film No. 7 with jojoba oil. On the left side—appearance of the film after film-forming gel preparation and application on the surface, on the right side—appearance of the film after 1 h.

During the research, castor oil was replaced with jojoba oil, trying to obtain high-quality, fast-drying films. The composition was 15% nitrocellulose, 24.7% ethanol, 51.9% ethyl acetate, and 6.4% jojoba oil. Visually, the gel containing jojoba oil was similar to gel 6, but the differences became visible after pouring it in a thin layer and observing the film formation process. After a few minutes, the film started to wrinkle, shrink at the center, and uncured gel remained at the edges. After removal from the surface, and trying to stretch the film, it was inelastic and started to turn white, so for further experiments jojoba oil was not used. The condition of the films is shown in Figure 2.

According to the results, the gels of formulations 5 and 6 formed films with optimal properties. They were selected for further studies after adding 2% of cinnamon leaf essential oil as an active antibacterial agent. Due to the rapid formation of films and excellent appearance, these products would be suitable for use by patients, and the transparency of films will allow for monitoring of the wound’s condition.

### 3.2. Results of Drying Time Test of Films

After selecting the most suitable composition, the drying time test of the films formed by the gels at 33–36 °C temperature was carried out. Drying time is a crucial factor in evaluating the quality of films. During the study, three (1–3) tests were performed by pouring the gels in a 0.8 mm layer and three (4–6) in a 1.5 mm layer. The drying of the formed films was assessed visually at the beginning—it was observed when the surface became no longer gel-like, and drying time was recorded with a timer. After that, a microscope slide was placed on top of it to check if the film was completely formed. Drying time results presented in Table 2 show that the film formation time for the gel layers of 0.8 mm was almost double for the fully prepared formulations compared to the controls: in both cases, control compositions dried faster (*p* < 0.05). A statistically significant difference (*p* < 0.05) was observed when comparing samples with the active substance—the films formed by the gels of formulation 6 dried faster than of formulation 5. On the contrary, three tests when casting 1.5 mm gel layer (tests marked with an * in Table 2) showed that both compositions needed more time to form films and the difference between the obtained data was not statistically significant (*p* > 0.05). Due to the data on the drying time of 0.8 mm thickness films, it could be assumed that the addition of essential oil to the composition slowed down the drying of the films, but comparing the drying of 1.5 mm thickness films in the same way, it was found that there is no statistically significant difference between drying times (*p* > 0.05). Researchers have found that films containing PVP/PVA materials dried about 3 min [26]. According to Velaga et al. [27], films prepared from hydroxypropyl methylcellulose and polyvinyl alcohol had various drying times depending on drying temperature: hydroxypropyl methylcellulose was 6.39, 4.65, and 3.62 min at 40, 60, and 80 °C, respectively, and the polyvinyl alcohol was 6.98, 4.18, and 3.03 min at 40, 60, and 80 °C, respectively. Different results were obtained by Tapia-Blacido et al. [28], who prepared the film from *Amaranthus cruentus* seed flour. The drying time of 80 ± 5 µm thickness films depended on drying conditions (temperature and relative humidity)—0 °C, 40% RH; 30 °C, 70% RH; 50 °C, 40% RH; 50 °C, 70% RH; 25.9 °C, 55% RH; 54.1 °C, 55% RH; 40 °C, 33.8% RH; 40 °C, 76.2% RH; and 40 °C, 55% RH. The fastest drying of film was obtained using 50 °C, 40% RH—4.2 h, and the slowest drying was at temperature 30 °C, RH 70%–14.6 h [28]. Longer drying time depends on lower solution evaporation because of lower temperature and higher humidity.

### 3.3. Results of Evaluation of the Surface and Homogeneity Properties of the Films

**Figure 3:**
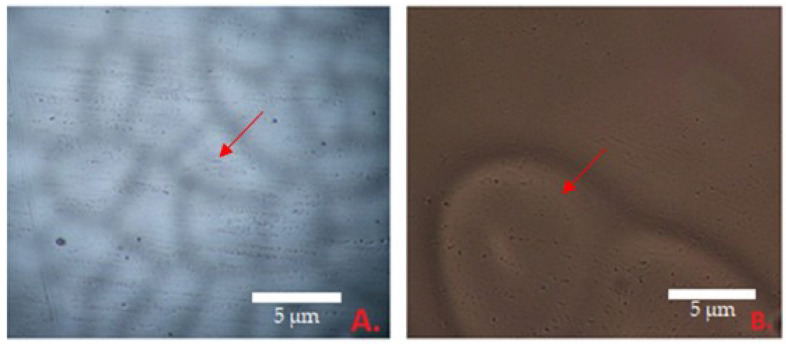
The surface of the film 40× magnification: (**A**) film No. 5; (**B**) film No. 5K (control compositions); arrows show the dimples of the film surface.

The morphological properties of the films were evaluated by microscopic analysis at 40 times magnification. Figure 3B shows the surface of the 5K film. The smallest particles were 3.17 µm wide, the largest—10.3 µm. Figure 3A shows the surface of composition 5. The smallest diameter of the particles was 8.8 µm, and biggest—23.93 µm. The average diameter of analyzed objects was 14.69 µm. To determine the effect of essential oil on the morphology of the films, control films were also evaluated. The average of particles is two times bigger than in composition 5 (6.14 µm).

**Figure 4:**
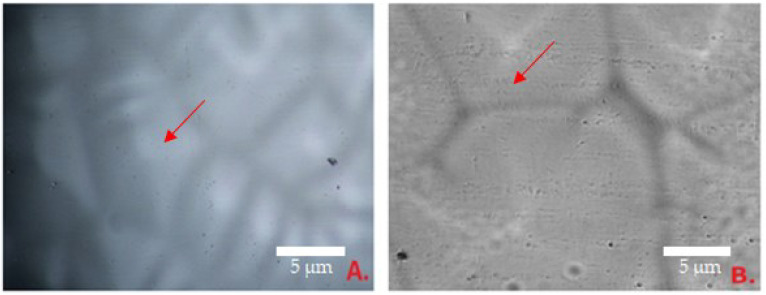
The surface of the film 40× magnification: (**A**) film No. 6; (**B**) film No. 6K (control compositions); arrows show the dimples of the film surface.

The diameter of the particles from formulation 6 are shown in Figure 4. Control formulation 6 is shown in B: the smallest particles of this sample were 2.20 ± 0.85 µm and the largest—12.44 ± 0.71 µm. The smallest particles of composition 6 (Figure 4A) were 8.21 ± 0.69 µm, the largest—20.2 ± 76 µm, and the average—14.12 ± 0.88 µm. The dimples of the formulation 6K films were significantly smaller than of formulation 6—the average diameter was 7.26 µm (Figure 4B).

According to the results, the dimples of films are very small. Comparing the averages of surface indentations of the films of the fifth and sixth compositions, the similarity of the diameters is noticeable, so it was not possible to single out the superior composition in this case. Adding the essential oil to the composition caused the formation of pits twice as large. Small bends are natural, indicating that volatile components were removed during the formation of the films. The films were formed qualitatively because none of the gels formed clumps when drying.

### 3.4. The Results of the Tests on the Adhesiveness and Adhesion of the Films to the Surface

**Figure 5:**
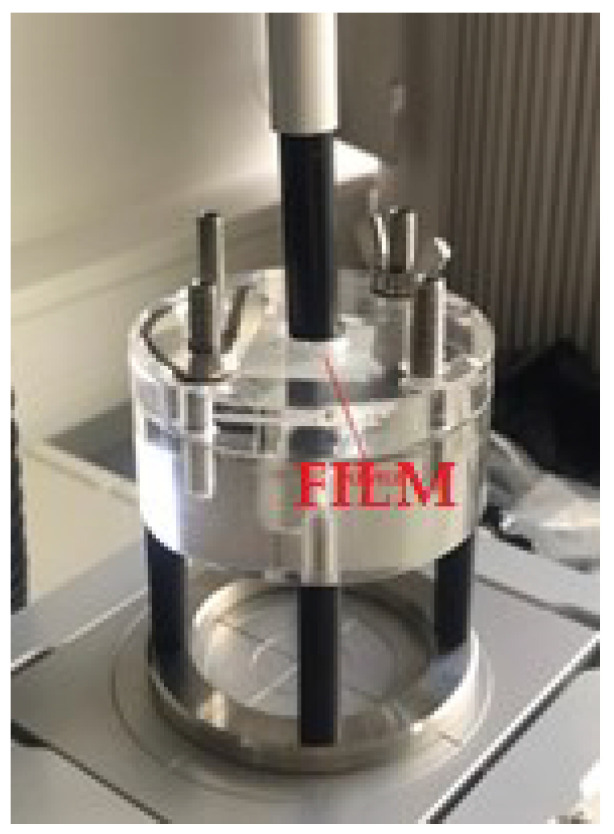
An apparatus to perform adhesion test for films.

The film surface adhesion test was based on the amount of force required to peel the surface of the texture analyzer stick from the film surface (Figure 5).

**Figure 6:**
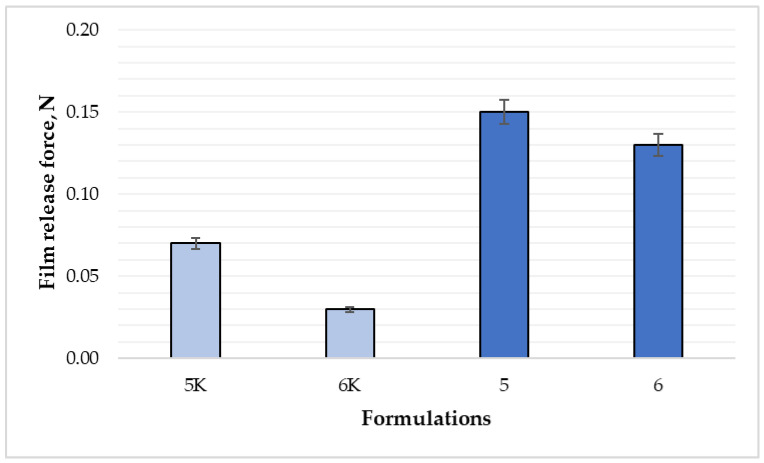
Comparison of film adhesion between formulations (n = 6). The composition values are given in Table 1. *p* < 0.05 between 5 and 5K; 6 and 6K compositions.

The tests showed a statistically significant difference between the averages of the force required to peel the film from the surface between the 5 and 5K composition films (0.15 N and 0.07 N, respectively) (*p* < 0.05). A statistically significant difference (*p* > 0.05) was found between the composition 6 and 6K. Figure 6 shows the film adhesion comparison between all formulations. Between compositions 5 and 6, adhesiveness remains very similar, so the difference is not significant (*p* > 0.05).

**Figure 7:**
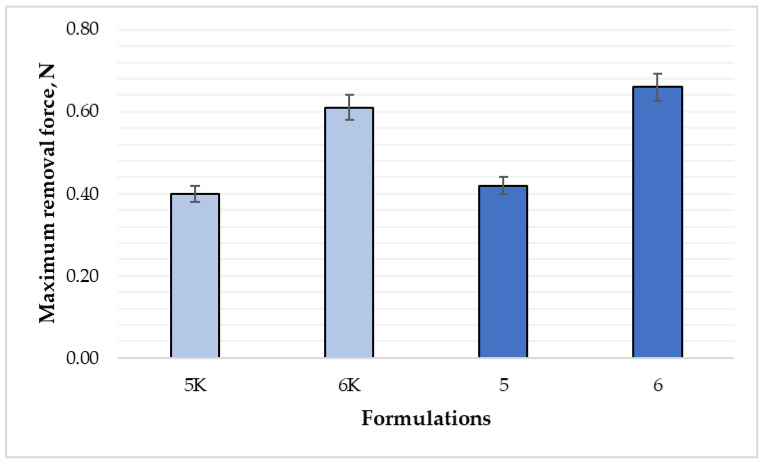
Comparison of film removal force by composition (n = 8). The composition values are given in Table 1. *p* < 0.05 between 6 and 6K; 5 and 6 compositions; *p* > 0.05 between 5 and 5K compositions.

**Figure 8:**
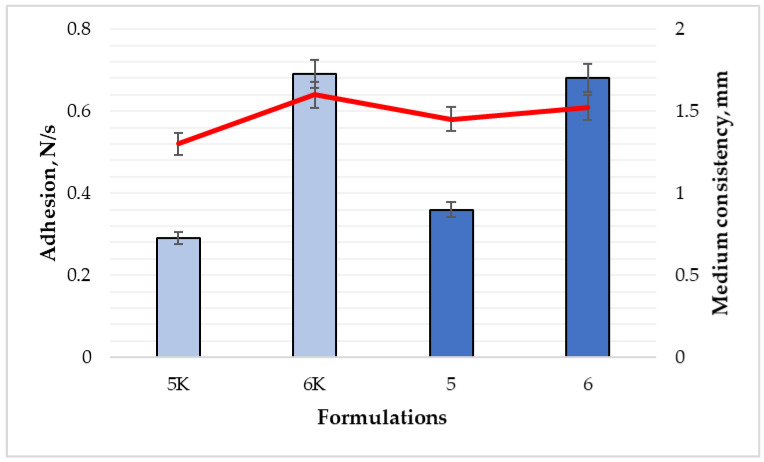
Comparison of adhesion and cohesiveness between formulations (n = 8). The composition values are given in Table 1. *p* < 0.05 between 5 and 5K compositions; *p* > 0.05 between 6 and 6K; 5 and 6 compositions.

Unlike surface adhesion, the inner surface of films must be sufficiently adhesive to adhere to the wound. To evaluate this property, a wound adhesion test was used, which shows three parameters—average film removal force peak, cohesiveness, and overall adhesion. The results of formulations 5 and 5K, in Figure 7 and Figure 8, revealed that there were no differences between maximum removal force and total surface adhesion results (*p* > 0.05), while the cohesiveness (indicated in red line Figure 8) was 1.45 mm for formulation 5, and 1.3 mm for formulation 5K.

For formulations 6 and 6K (Figure 7), the results showed a statistically significant difference only for maximum film removal force (0.66 N and 0.61 N, respectively) (*p* < 0.05). The data of cohesiveness and adhesion were similar, and the difference was not significant (*p* > 0.05). According to the results, essential oil could affect the adhesion to the surface of the films. The adhesion test results of nitrocellulose films are quite like sodium alginate films. Fiume et al. evaluated the effect of protein content on the adhesion properties of films: removal force peak was 0.49 N, adhesion—0.15 N/s, and cohesiveness—1.6 mm [29]. This data corresponded to the 5K composition film results.

According to the obtained results, the addition of cinnamon leaf essential oil increased the surface adhesion of the films but did not affect the wound adhesion parameter. The adhesion of formulation 5 increased about two times, and formulation 6—more than four times (Figure 8). The internal adhesion test showed that the gels of the sixth formulation formed films with a total adhesion and maximum average removal force almost twice that of the 5th formulation. Coherence was not significantly different. These results allow us to confidently assert that the sixth gel composition, which contains more nitrocellulose, would adhere more strongly to the damaged area, thus forming a reliable barrier. Additionally, it would be more convenient to apply on the surface of the wound, because it would not stick to clothes, bedding, or other surfaces.

### 3.5. Results of Film Flexibility Tests

**Figure 9:**
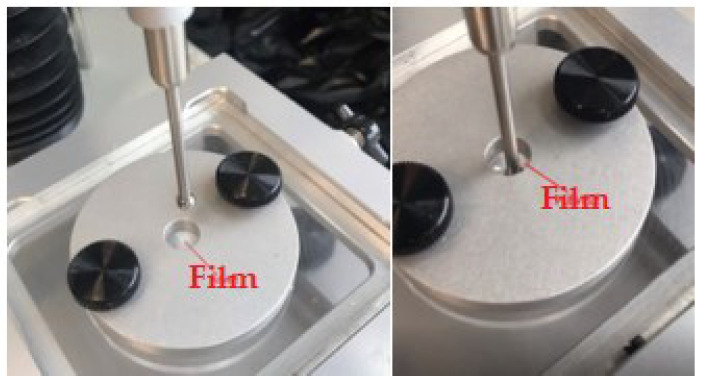
Pressure resistance test for films.

Two tests were performed to determine the flexibility of the films. The differences in the flexibility of the films of compositions 5 and 6 and control compositions were tested. The purpose of the compression test was to find out the maximum force that can be pressed down on the film before it tears (Figure 9).

**Figure 10:**
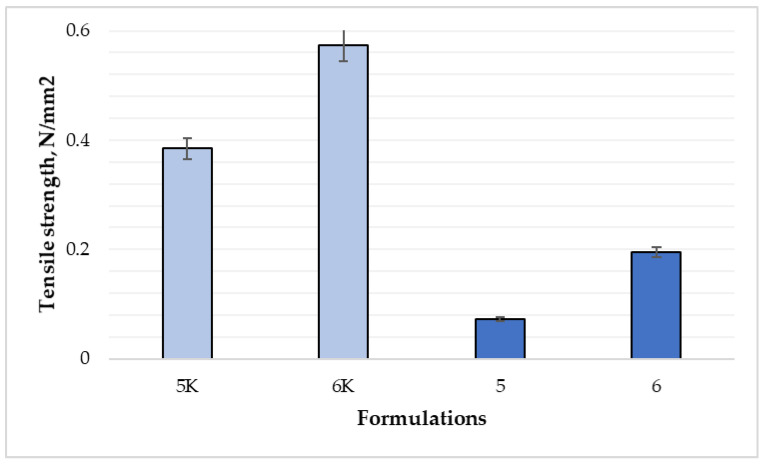
Comparison of tensile strength by composition (n = 6). The composition values are given in Table 1. *p* < 0.05 between 5 and 5K; 6 and 6K; 5 and 6 compositions.

Flexibility properties of the films were compared (Figure 10). The difference between control compositions, 5 and 6 compositions were statistically significant (*p* < 0.05). Tensile strength of 6K was the highest of all the samples—0.5735 N/mm^2^, sample 5K—0.3846 N/mm^2^. Data show that the tensile strength of film 6 was 2.7 times higher than film 5 (0.1948 and 0.0728 N/mm^2^, respectively). The addition of essential oil of cinnamon leaves and higher concentration of nitrocellulose strongly reduced the elasticity and strength of the films. Momoh et al. [17] claimed that the optimal properties of the films are achieved when there is a balance between strength (brittleness) and elasticity (flexibility)—tensile strength of alginate film was 6.12 ± 0.11 N/mm^2^. According to results from Marangoni Junior et al. [30], a higher concentration of film-forming gel additives, such as propolis extract and silica, ensured higher tensile strength of sodium alginate films: only sodium alginate film—12.9 MPa, sodium alginate and 3% propolis extract—16.5 MPa, sodium alginate, 3% propolis extract, and 5% silica—18.2 MPa and sodium alginate, 3% propolis extract, and 10% silica—19.6 MPa. Nitrocellulose films have lower tensile strength because of different polymer (nitrocellulose vs. sodium alginate) structure and different compounds used in composition (castor oil, CLEO vs. propolis extract, silica). According to other studies, tensile strength could increase after adding higher concentrations of polymers, plasticizers, and other bonding compounds.

**Figure 11:**
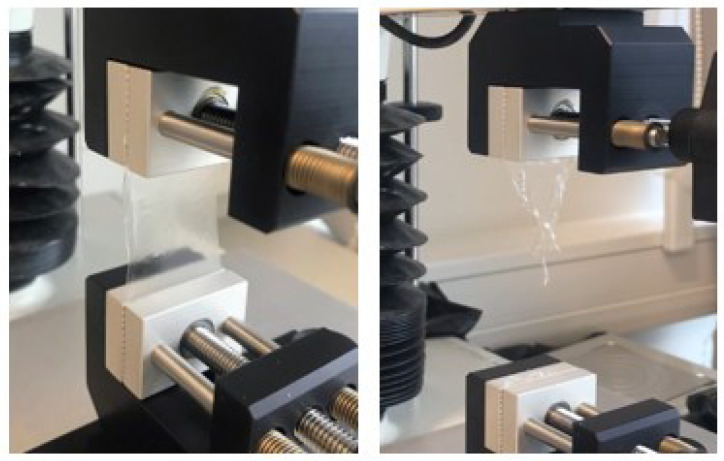
Film before (left) and after (right) tensile test.

The second test for assessing flexibility is tensile. In this test, the film was attached to the clamps from two sides and the upper clamp was pulled up until it teared (Figure 11).

**Figure 12:**
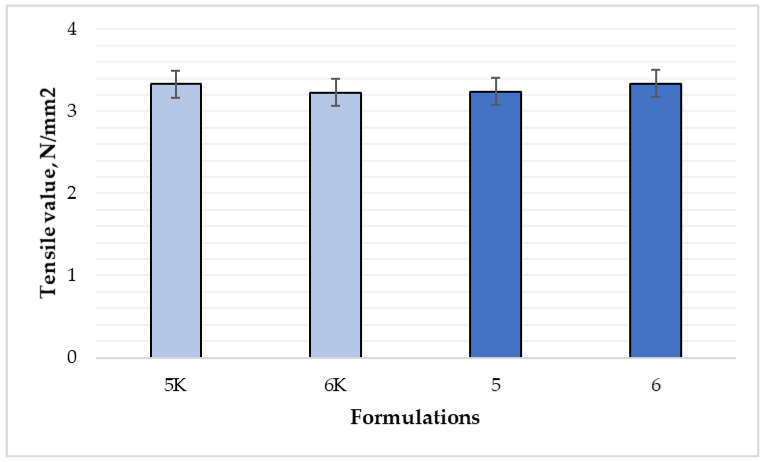
Comparison of tensile value of films by composition (n = 6). The composition values are given in Table 1. *p* < 0.05 between 5 and 5K; 6 and 6K; 5 and 6 compositions.

The results showed (Figure 12) statistically significant differences in tensile values between all the tested films (*p* < 0.05). The tensile value of formulations 5 and 5K was 3.24 N/mm^2^ and 3.33 N/mm^2^, respectively, for formulations 6 and 6K—3.34 N/mm^2^ and 3.23 N/mm^2^, respectively.

The results of the films’ flexibility showed that the resistance and tensile tests do not correlate and the effect of the essential oil the elasticity of the films could not be proven. Furthermore, due to the conflicting results of the two tests, it would not be correct to claim that the flexibility of composition 6 is better than composition 5.

### 3.6. Water Vapor Penetration Test Results

**Figure 13:**
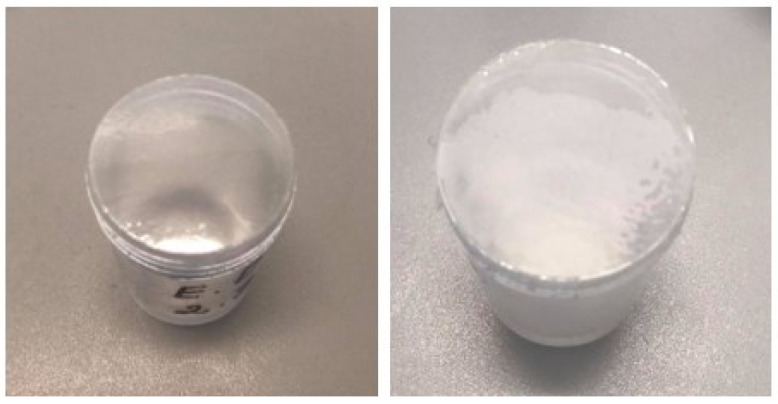
Water vapor transmission rate (WVTR) test. Films before (left) and after (right).

The water vapor permeability test is an important indicator that shows how the film is able to retain moisture in the wound. During the test, samples were placed in an incubator and kept for 24 h. After 24 h, the samples were analyzed and weighed. The subjects were slightly pale after the test. This was most likely due to the ability of nitrocellulose to interact with water molecules and turn from colorless to white (Figure 13).

**Figure 14:**
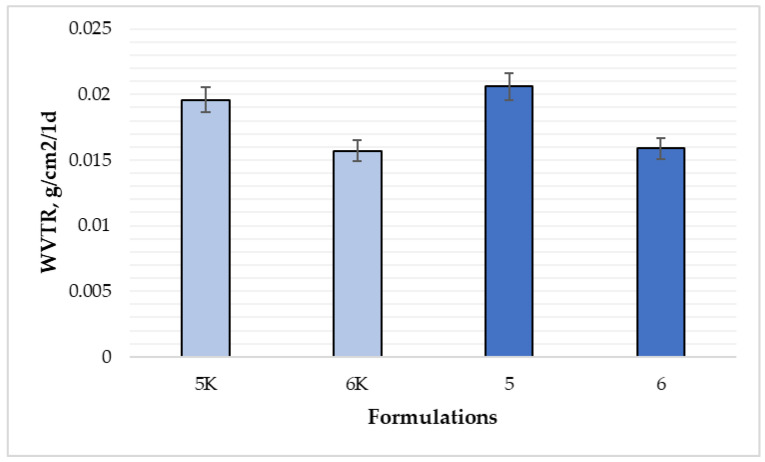
Comparison of average water vapor permeation through the film by composition (n = 3). The composition values are given in Table 1. *p* < 0.05 between compositions 5 and 6; *p* > 0.05 between 5 and 5K; 6 and 6K compositions.

Figure 14 shows the results of WVTR for compositions 5, 5K, 6, and 6K. Film formulation 5K was able to transmit 0.0196 g of water vapor per 1 cm^2^ per day, and formulation 5K—0.0206 g (*p* < 0.05). Formulations 6 and 6K transmitted less water vapor than formulations 5 and 5K. There was no difference in WVTR between 6 and 6K films (*p* > 0.05). Results from Maragoni Junior et al. [30] show that, the addition of propolis extract and silica provided a decrease in WVTR: the control film (708.7 ± 60.9 g m^−2^ day^−1^) and the film containing the highest concentration of silica (10%) (595.2 ± 3.5 g m^−2^ day^−1^) (reduction about 16% in this variable). Furthermore, the authors found that the reduction of WVTR may have been influenced by the tendency of increasing film thickness, particularly at the highest filler load [30]. Tapia-Blacido et al. [28] found that WVTR of films prepared with sorbitol is lower than that of glycerol-containing films: the better water vapor barrier properties of films containing sorbitol as plasticizer compared with those of the films containing glycerol might be because sorbitol is less hygroscopic.

According to obtained data, all film formulations are very poorly permeable to moisture, which may cause discomfort to patients. The moisture that accumulates in the wound will not be removed, causing the tissues to swell, stretch, and not heal. The amount of nitrocellulose in the composition affected the transmission of water vapor, while the addition of essential oil had no impact on it.

### 3.7. Swelling Test Results of the Films

**Figure 15:**
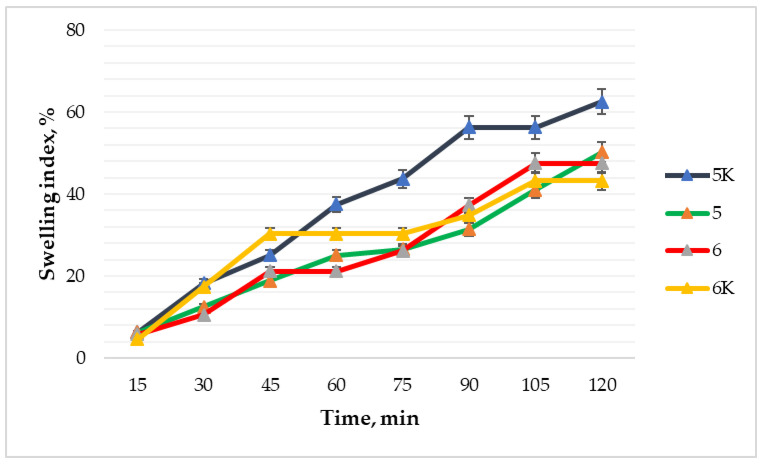
Comparison of swelling index, % variation with time (n = 3). The composition values are given in Table 1.

A test that analyzes film ability to absorb wound exudate was carried out for 2 h by weighing the films, which had previously been dried with filter paper, every 15 min. Figure 15 shows that formulation 6 maintained the lowest swelling index (4.55%) during the first 15 min of the test. At 45 min of the test, the percentage of wound fluid absorption increased to 30.3%. Then the swelling index stopped for a while and only between the 75 and 90 min of the study it started to increase again. The highest swelling index was after 2 h for composition 5K. Comparing compositions 5 and 5K, it was observed that the increase in the swelling index of both took place evenly, but composition 5 was less swollen. Only a small difference in the level of absorption of wound fluid was observed between compositions 6 and 6K. The composition of 6K initially had only a 1.25% higher swelling index than composition 6 and the difference increased to just 4.32% over time. Thus, the 6 formulation showed the lowest swelling ability after the 2 h test. Fan et al. [31] data matches our results because the swelling rate decreases with increasing the concentration of nitrocellulose, which decreases from 19.86% at 2% solid content to 3.13% at 16% solid content.

Man et al. [12] studied the properties of sodium alginate films and found that for effective wound healing, the films must be able to absorb a large amount of fluid secreted by the wound: the swelling index of the film with sodium alginate was 85% after 2 h.

According to the results, the control formulations showed slightly better wound fluid absorption capacity, suggesting that cinnamon leaf essential oil slightly reduced the swelling properties of the films. In the compositions with the active substance, the swelling index was 43.18 and 50.2% (compositions 6 and 5, respectively). The lower swelling of the formulation 6 could be explained by the higher amount of nitrocellulose leading to an inferior interaction with water.

### 3.8. Microbiological Test Results

**Figure 16:**
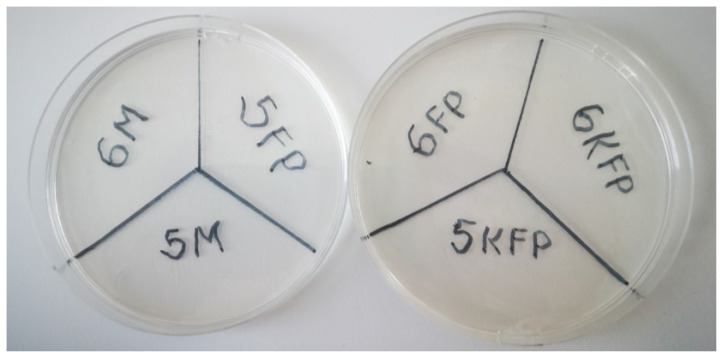
No bacteria growing was detected on film-forming gels. M—film-forming gels, stored for a month at 38 ± 2 °C; FP—freshly prepared film-forming gels; K—control formulations.

Six samples were tested in this test for the microbiological contamination—gels of formulations 5 and 6 stored for a month at 38 ± 2 °C, freshly prepared gels of formulations 5 and 6, and freshly prepared control samples of formulations 5 and 6 without essential oil. *S. aureus* and *P. aeruginosa* bacteria were searched for in the samples mentioned. The obtained results confirmed the expectations—no bacteria were detected in any of the samples (Table 3, Figure 16). The essential oil may not have been the most important factor in the absence of bacteria. Control formulations without the active substance also inhibited the growth of microorganisms in the gels. The absence of bacteria in the gels proves that the product is suitable for wound treatment, and it does not cause soft tissue infections, such as purulent/non-suppurative Impetigo, ecthyma gangrene, staphylococcal scalded skin syndrome (SSSS), rosacea, etc. [32]. Mu et al. [1] found that nanopores formed by nitrocellulose inhibit the penetration of bacteria through the film, but this microbiological study could indicate that the gelling substance also has microorganism-killing properties, and the essential oil of cinnamon leaves was not the most important factor determining the proper microbiological quality of the gel.

### 3.9. Stability Test Results

The stability of the film-forming gels and films of formulations 5 and 6 was tested for 28 days at 38 ± 2 °C. Monitoring changes in stability took place on the day of production, and on days 7, 14, 21, and 28. The color, integrity, and surface of the films did not change during stability test (Table 4). The films remained intact, there were no visible cracks or thinning, no change of color. Film-forming gels after 28 days had no color, odor, viscosity, texture changes (Table 5). Film-forming gels based on various grades hydroxypropyl methylcellulose (HPMC) remained stable for 3 months at 40 °C and 75% RH for accelerated stability and at 4 °C in the fridge [33] and hydroxypropyl cellulose-based film-forming gels—6 months in the same conditions [16]. Bharti et al. [34] used HPMC as the base for film-forming gels to prepare films with buspirone hydrochloride nanoparticles. After 90 days at 25 °C and 60% relative humidity there was no difference seen in the physical appearance, surface pH, and disintegration time of films [34].

### 3.10. Evaluation of the pH Value of Film-Forming Gels

**Figure 17:**
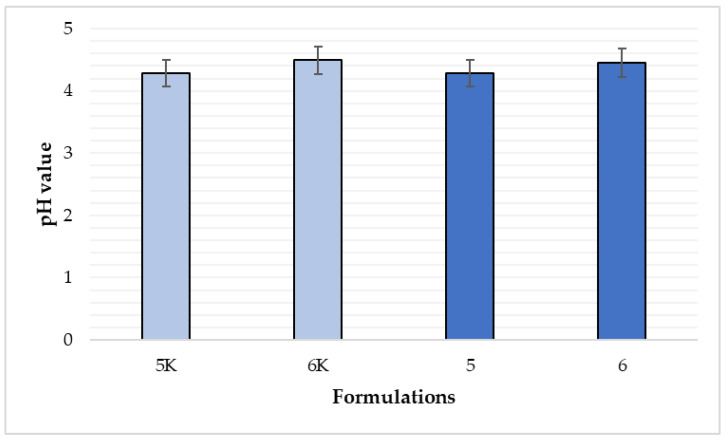
Comparison of pH values by composition (n = 6). The composition values are given in Table 1. *p* < 0.05 between compositions 5 and 6.

The normal pH value of human skin is slightly acidic. It is an important factor for the maintenance of normal skin condition and microflora [35]. In the case of chronic wounds, the pH indicator becomes more alkaline (between 7.15 and 8.1). It has been shown that if pH value is higher, the wound healing is slower [36]. To make sure that the product will not irritate the skin and at the same time promote the healing of skin, pH value of film-forming gels was determined. After making aqueous solutions of film-forming gels, six pH meter measurements were performed for each formulation. Formulation 5 showed pH value of 4.28, while the pH value of formulation 5K was 4.29. Formulation 6 had a pH value of 4.45 and formulation 6K had a pH of 4.49. These slight differences between the control and non-control formulations indicated that the essential oil of cinnamon leaves does not affect the pH value of the formulation. Comparing the differences in the pH values of formulations 5 and 6 (Figure 17), it shows that the pH value of formulation 6 is slightly more alkaline and the difference between formulations 5 and 6 is statistically significant (*p* < 0.05). Both products have a pH value below 5 and are therefore considered suitable for use on the skin.

### 3.11. Results of Qualitative and Quantitative Analysis of Cinnamon Leaf Essential Oil

**Figure 18:**
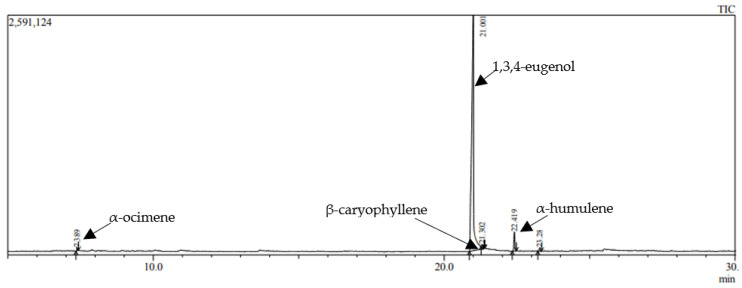
A GC-MS chromatogram of the cinnamon leaf essential oil.

Qualitative and quantitative analysis of cinnamon leaf essential oil (*Cinnamonum zeilanicum*) by GC-MS showed that the product contains 4 components. As shown in the chromatogram (Figure 18), the highest peak was 1, 3, 4—eugenol (94.14%).

Other components’ presence was significantly less—3.96% α-humulene, 0.57% α-ocimene, and 0.55% β-caryophyllene. Eugenol has anesthetic, antioxidant, anti-inflammatory, antimicrobial activities [37]. Eugenol may give to the film-forming gel excellent antibacterial and anti-inflammatory properties, and applying the product to the wound would improve the healing processes.

## 4. Conclusions

Physico-chemical and mechanical analyses of film-forming gels and films is important for the creation of high quality and suitable-for-use dermatological films. A range of factors could impact the formation of films, appearance, mechanical properties, stability, and release of active ingredients. According to the findings of previous research, films containing nitrocellulose have proven to be suitable for use as a delivery form due to its high physico-chemical, mechanical, biopharmaceutical properties and cosmetic attractiveness. Nitrocellulose based films showed acceptable drying rate, adhesion, flexibility, tensile strength, cohesiveness, swelling, water vapor penetration, and pH value. The results of these parameters confirm the ability to use these films on skin. This study has shown that the concentration of nitrocellulose, castor oil, and essential oil could impact the drying ratio, mechanical properties, and other parameters. The results of the study have provided fundamental insights into the preparation of film-forming gels and films that are highly relevant for diverse industries such as pharmaceutical, food, cosmetics, and agriculture. Future work will focus on extending the applicability of hydrophilic and hydrophobic polymers cast from aqueous and organic solvents, investigating the effects of the thickness of the film, different drying conditions, active substance release in vitro, anti-inflammatory, and antibacterial effects on the wounds.

## Figures and Tables

**Table 1:** Composition of film-forming gels.

Ingredients	Formulations								
	5 K	6 K	1	2	3	4	5	6	7
Nitrocellulose	13.4%	15%	11%	15.4%	15.4%	16%	13.4%	15%	15%
Ethanol (96%)	26.1%	24.7%	25.7%	24.6%	24.6%	24.7%	26.1%	24.7%	24.7%
Ethyl acetate	52.2%	51.9%	51.3%	54.8%	51.7%	51%	52.2%	51.9%	51.9%
Castor oil	6.3%	6.4%	10%	4%	6.3%	6.3%	6.3%	6.4%	-
Jojoba oil	-	-	-	-	-	-	-	-	6.4%
CLEO	-	-	-	-	-	-	2%	2%	-

CLEO—cinnamon leaf essential oil (active ingredient), K—control.

**Table 2:** Results of the drying rate of films with different compositions.

Tests	Formulations			
	5 K	6 K	5	6
1 test *	1 min 04 s	0 min 56 s	2 min 36 s	1 min 27 s
2 test *	1 min 08 s	0 min 44 s	2 min 14 s	1 min 25 s
3 test *	1 min 25 s	0 min 52 s	1 min 58 s	1 min 15 s
4 test **	2 min 51 s	2 min 20 s	2 min 47 s	2 min 55 s
5 test **	2 min 43 s	2 min 12 s	3 min 02 s	2 min 20 s
6 test **	2 min 36 s	2 min 10 s	3 min 10 s	2 min 30 s

*—gel poured 0.8 mm layer; **—gel poured 1.5 mm layer; K—the control formulation, which does not contain cinnamon leaf essential oil.

**Table 3:** Results of film-forming gels microbiological analysis.

Bacteria Strains	Formulations					
	5M	6M	5FP	6FP	5KFP	6KFP
*S. aureus*	-	-	-	-	-	-
*P. aeruginosa*	-	-	-	-	-	-

M—film-forming gels, stored for a month at 38 ± 2 °C; FP—freshly prepared film-forming gels; K—control formulations; —no bacteria was detected.

**Table 4:** The stability test for films No. 5 and No. 6.

Days	Film Formulation 5	Film Formulation 6
Production day (0)	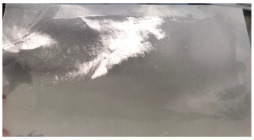	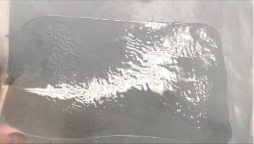
7	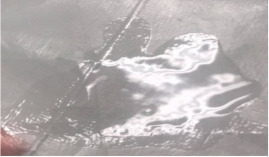	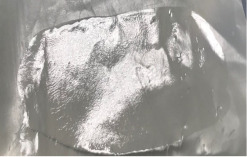
14	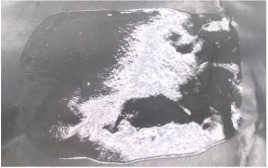	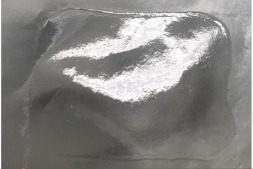
21	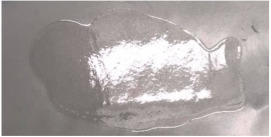	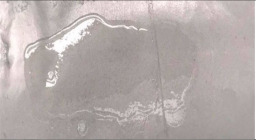
28	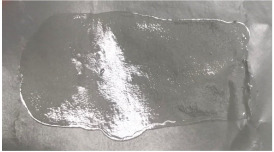	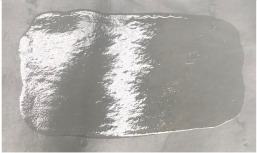

**Table 5:** Status of the control and non-control gels formulation before and after the stability study.

	Control	With CLEO
Before test (production day)	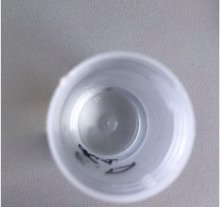	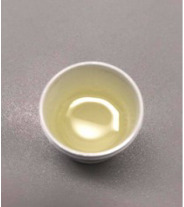
After test (28 days)	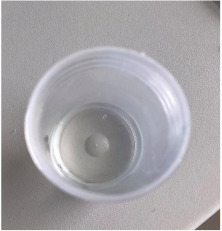	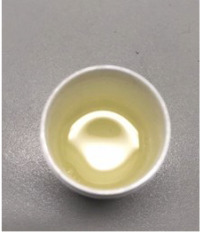

CLEO—cinnamon leaf essential oil (active ingredient).

## Data Availability

Not applicable.
